# Epidemiological Factors Associated With *Caligus rogercresseyi* Infection, Abundance, and Spatial Distribution in Southern Chile

**DOI:** 10.3389/fvets.2021.595024

**Published:** 2021-08-20

**Authors:** Manuel Lepe-López, Joaquín Escobar-Dodero, Daniel Rubio, Julio Alvarez, Natalia Zimin-Veselkoff, Fernando O. Mardones

**Affiliations:** ^1^PhD Program in Conservation Medicine, Facultad de Ciencias de la Vida, Universidad Andres Bello, Santiago, Chile; ^2^Facultad de Ciencias de la Vida, Centro de Investigación para la Sustentabilidad, Universidad Andres Bello, Santiago, Chile; ^3^Department of Veterinary Population Medicine, University of Minnesota, St. Paul, MN, United States; ^4^EPIVET Analysis & Solutions, Santiago, Chile; ^5^Centro de Vigilancia Sanitaria Veterinaria (VISAVET), Universidad Complutense, Madrid, Spain; ^6^Departamento de Sanidad Animal, Facultad de Veterinaria, Universidad Complutense, Madrid, Spain; ^7^Escuela de Medicina Veterinaria, Facultad de Agronomía e Ingeniería Forestal, Facultad de Ciencias Biológicas, Facultad de Medicina, Pontificia Universidad Católica de Chile, Santiago, Chile

**Keywords:** sea lice, *Caligus rogercresseyi*, salmon farming, host-density, integrated pest management

## Abstract

Sea lice (*Caligus rogercresseyi*) are external parasites that affect farmed salmonids in Chile, and the scale of their sanitary and economic impact cannot be overstated. Even though space–time patterns suppose parasite aggregation, specific locations related to different infestation levels, as well as their associated factors across the geographic range involved, had not been investigated as of the writing of the present article. The understanding of the effects and factors entailed by the presence of *C. rogercresseyi* may be deemed a key element of Integrated Pest Management (IPM). In the present study, the multivariate spatial scan statistic was used to identify geographic areas and times of *C. rogercresseyi* infestation and to estimate the factors associated with such patterns. We used official *C. rogercresseyi* monitoring data at the farm level, with a set of 13 covariates, to provide adjustment within the analyses. The analyses were carried out for a period of 5 years (2012–2016), and they included three fish species (*Salmo salar, Oncorhynchus mykiss*, and *Oncorhynchus kisutch*) in order to assess the consistency of the identified clusters. A retrospective multinomial, spatial, and temporal scan test was implemented to identify farm clusters of either of the different categories of *C. rogercresseyi* infested farms: baseline, medium, and high, based on the control chemical threshold established by the health authority. The baseline represents adequate farm performance against *C. rogercresseyi* infestation. Then, production and environmental factors of the medium and high infestation farms were compared with the baseline using regression techniques. The results revealed a total of 26 clusters (*p* < 0.001), of which 12 correspond to baseline, 1 to medium, and the remaining 13 to high infestation clusters. In general, baseline clusters are detected in a latitudinal gradient on estuarine areas, with increasing relative risks to complex island water systems. There is a spatial structure in specific sites, north of Los Lagos Region and central Aysén Region, with high infestation clusters and epidemic peaks during 2013. In addition, average weight, salmon species, chemotherapeutants, latitude, temperature, salinity, and year category are factors associated with these *C. rogercresseyi* patterns. Recommendations for an IPM plan are provided, along with a discussion that considers the involvement of stock density thresholds by salmon species and the spatial structure of the efficacy of chemical control, both intended to avoid the advance of resistance and to minimize environmental residues.

## Introduction

In the Southern Hemisphere, *Caligus rogercresseyi* is the external parasite affecting most farmed salmonid species (*Salmo salar, Oncorhynchus mykiss*, and *Oncorhynchus kisutch*), with enormous sanitary and economic costs. In Chile, the second largest producer worldwide and the largest producer of farmed salmonids in South America, the cost unit of Atlantic salmon (*S. salar*) increases by an average price of US$ 1.4/kg, due to control measures against *C. rogercresseyi* (1). As a result of Chilean industry production, 953,296 tons/year at the end of 2019 (where *S. salar* represented 75%, *O. mykiss* 9%, and *O. kisutch* 16% of the total), the cost of these measures amounted to ~US$ 1 billion (2). The life cycle of this copepod presents eight developmental stages. Three are planktonic, and the remaining five are parasitic (3). The non-parasitic stages include two naupliar stages and one copepodid or parasitic stage. The parasitic stages include four chalimus stages attached by the frontal filament to the fish, mounting male and female adults. The chromosome-scale whole genome sequence has been reported (4). Like other parasitic copepods of the Caligidae family (commonly named as sea lice), *C. rogercresseyi* feed on the mucus and tissue of the host, impairing both feed conversion and carcass quality, thus affecting the commercial value of the product (5). Atlantic salmon with *C. rogercresseyi* present skin abrasions, followed by osmotic problems, physiological stress, hematological alterations, reduced appetite, weight loss due to energy demand, and secondary bacterial or viral infestations (6–8).

In 2007, the Chilean National Fisheries Service (Sernapesca), acting in its role as a point of reference for the technical surveillance of the Salmon Technical Institute (INTESAL), implemented a national surveillance program for the monitoring of the abundant presence of *C. rogercresseyi* (9). This monitoring consists in taking random samples of 10 fish from four cages in each salmon farm, counting the parasitic stages and dividing those counts by the number of fish. From this sample, trained personnel carry out counts of chalimus, including male and non-gravid female adults, and adult gravid female stages of *C. rogercresseyi*. A cross-sectional retrospective analysis of 2007 data allowed to establish that *C. rogercresseyi* is widely distributed in Region X [Los Lagos] and Region XI [Aysén], with a decreasing epidemiological case occurrence in farms located in southern areas (10). Field studies suggest the influence of *C. rogercresseyi* infestation, based on the immune response of the fish host (*S. salar* and *O. mykiss* are most susceptible than *O. kisutch*), water temperature (biological cycle develops between 4 and 17°C), water salinity (survival decays <25 ppt), fish weight (host density), juvenile parasite count (the mucus covering on the fish protect these stages), and the number of chemical control events (3, 11, 12). Subsequently, two analyses of risk factors, based on data from 2006 to 2007, displayed parasitic persistence (sea lice counts > 11) associated with stocking density (over 22 kg m^−3^), the season of the year (increasing in autumn), depth of water at the farm and cage depth location (>50 m), geographical zones (there is a link between south areas and lower infestation), and chemical control performed 1 month before sampling (13, 14). Recently, a study using 7 years of Sernapesca regulatory data (between 2011 and 2017) suggests that the initial weight of smolt at seawater stocking (>156 g), cumulative degree-days (5,547 degree-days for Atlantic salmon, 3,411 degree-days for rainbow trout, and 2,630 degree-days for coho salmon), high fish density (odds ratio of 0.85), fish species (odds ratio of 1.50 for rainbow trout compared to Atlantic salmon), region (odds ratio of 0.65 for Region XI compared to Region X), and longitude (lower abundance at easterly locations) are associated factors with sea lice abundance (15).

After the sanitary crisis of infectious salmon anemia (virus ISA, reported by Sernapesca) in 2007 and new sanitary regulations enacted in 2012, salmon farming in Chile has been in constant expansion, including more than ~600 farms and adding Region XII [Magallanes] as a new geographical area (16, 17). To synchronize operating and rest activities, geographical regions X [Los Lagos], XI [Aysén], and XII [Magallanes] have been divided into ~60 epidemiological neighborhoods across different production and environmental conditions (2). The farms are active at dissimilar times, stocking different species of salmonids (*S. salar, O. mykiss*, and *O. kisutch*) and rotating active ingredients for sea lice chemical control. According to the dynamics of infectious diseases (18), *C. rogercresseyi* patterns are heterogeneous in space and time. A recent study, limited to three epidemiological neighborhoods with a persistent history of *C. rogercresseyi* infestation on northern Region X [Los Lagos] during 2012–2015, suggests temporal patterns with high peaks in one specific site exclusively during 2013 (19). Space–time patterns suppose parasitic aggregation across the geographic area occupied by salmon farming, with an increase in risk around predetermined sites. Also, the genetic analysis of *C. rogercresseyi* along a latitudinal range (40°-52°S) in southern Chile proposes genetic homogeneity, morphometric variability among sites, and longest parasites from >52°S (20). However, location areas associated with different infestation levels of *C. rogercresseyi* throughout three regions of Chile had not been investigated yet.

The effort to reduce *C. rogercresseyi* infestation on farmed salmonids in Chile is centered on the use of chemical control, either in baths or in feed. Historically, from 1999 to 2007, emamectin benzoate was used exclusively in the control of sea lice, resulting in loss of sensitivity to this chemical (21). Later, peroxide hydroxide and deltamethrin were approved for use in 2007. Approval was also granted for the use of diflubenzuron in 2009, cypermethrin in 2010, azamethiphos in 2013, lufenuron in 2017, and hexaflumuron in 2019 (22). Several investigations in loss of sensitivity, resistance, and chemical control failure suggest that frequently repeated exposure to these ingredients could change *C. rogercresseyi* molecular mechanisms of detoxification, transport the drugs along the biological membrane, and limit the ability to penetrate the copepod cuticle (21, 23–29). Biological control methods are being explored in Chile, with no significant effect either on the fecundity or survival of *C. rogercresseyi* (30).

In Chile, the dynamics of *C. rogercresseyi* maintain persistent levels of occurrence, which are associated with production practices shared by different farms and companies (fish stock densities). In the marine environment, different groups of farms cultivate a large number of susceptible fish hosts at the same time, becoming a source of infestation to neighboring farms and to themselves in a restricted spatial area (31). The spatial structure of *C. rogercresseyi* dynamics has been approached exclusively to evaluate the effect of chemical control, identifying a distance-dependent cluster response mediated by unknown factors (24, 25, 29). In addition, in most salmon-producing regions, local authorities encourage the adoption of Integrated Pest Management (IPM) and the exploration and use of other non-chemical measures to reduce sea lice occurrence levels (32, 33). The exploration of the spatial and temporal patterns of *C. rogercresseyi* could reduce potential interactions with areas susceptible to infestation, serving as an IPM approach in the marine environment of Chile. Recently, during 2017, a *C. rogercresseyi* outbreak was identified in the Magallanes Region (south 49°16′S), an area that includes only 10% of Chilean salmon production, and where supposedly the water temperature and salinity conditions are not ideal to the parasite life cycle (10, 13). If the IPM objective is to isolate susceptible farms from infested farms, then the problem of management centers on minimizing interaction in space and/or time within *C. rogercresseyi* sites of infestation. On the other hand, if the IPM objectives are to include the role of water currents and to reduce the transport of the planktonic stages, then the management of the problem must take into account the influence of this factor in different locations and/or times across the study area over the annual rainfall trend.

Effective IPM plans must be adapted to suit the regionally varying environmental and biological factors that affect the intensity of parasitic infestations. For example, the increase of superficial water temperature can accelerate the maturation of *C. rogercresseyi* eggs, taking 45 days at 10.3°C, and only 18 days at 16.7°C in experimental conditions (3). Besides, copepodid survival time is 30 and 44% longer than at 15 and 20°C (34). Low salinity concentration due to freshwater discharge from rivers reduces the survival rate of parasitic stages (35). In addition, the time and magnitude of river discharge vary during the seasons, depending on rainfall patterns and the influence of salinity concentrations. The understanding of risk factors such as temperature and salinity can be another tool in the development of an IPM plan for sea lice in southern Chile.

The space–time occurrence of a disease can be addressed using cluster analysis techniques. Clustering is a term used to describe the aggregation of disease, in both specific locations and time windows, to understand different events of interest (36). In this context, we are using local clustering methods for the detection of the locations and times of different *C. rogercresseyi* levels in order to understand the factors that could influence these patterns. We are interested in the study of three *C. rogercresseyi* levels of infestation, based on the parasitic threshold proposed by the health authority in Chile (if a farm reports *C. rogercresseyi* gravid female counts > 3, it is mandatory to apply chemical control to reduce loads). Therefore, we propose a multinomial modeling process. This includes infestation levels below the threshold, around the threshold, and infestation levels above the sanitary threshold to explore clustering patterns. The identification of space–time clustering of different infestation levels of *C. rogercresseyi* allows us to make comparisons that could inform the targeted investigation of the factor associated, as well as support the understanding of how to achieve better management concerning *C. rogercresseyi*. The objectives of this study were to identify geographic areas and times of *C. rogercresseyi* infestation levels throughout every salmon farming area in Chile by space–time cluster outcome and to estimate the factors associated with such patterns.

## Materials and Methods

### Study Area

**Figure 1:**
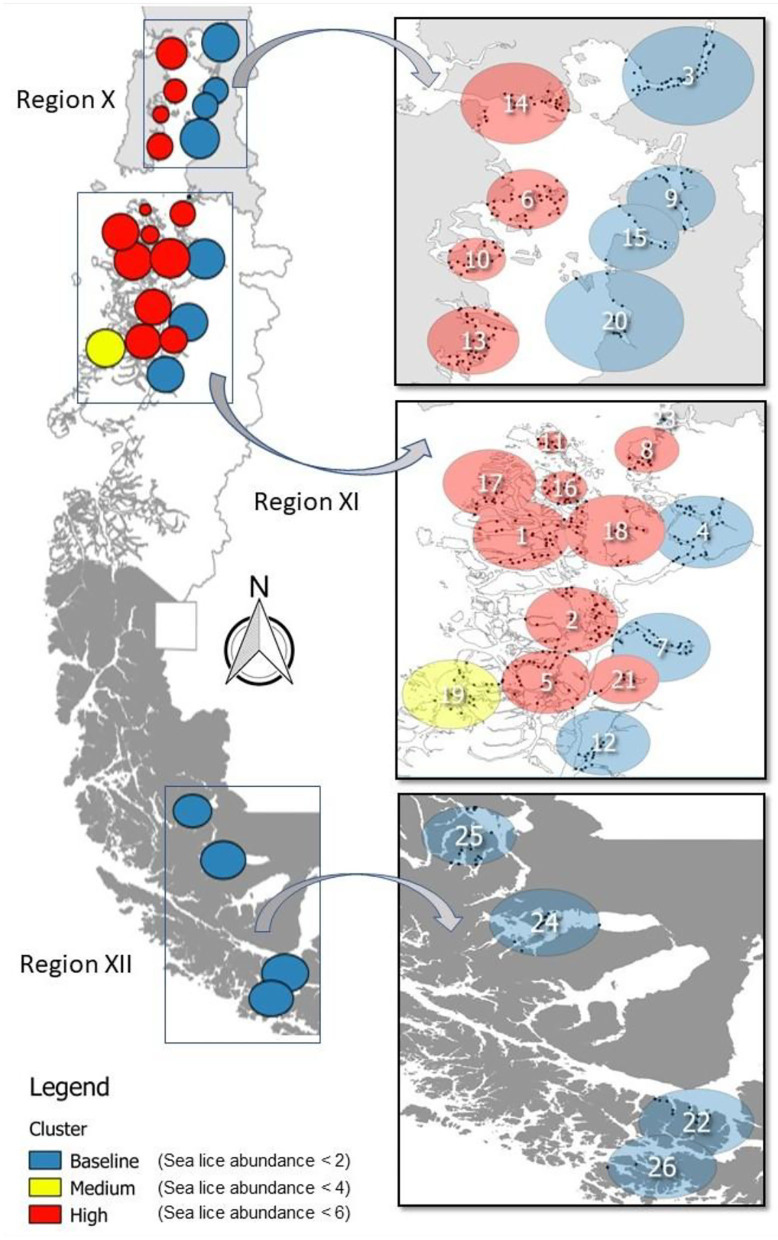
Production area occupied by salmon farming Chile (left) covers three geographic regions during 2012–2016: Region X [Los Lagos], Region XI [Aysén], and Region XII [Magallanes]. Clusters resulting from a multinomial space–time SaTScan model are identified by the *C. rogercresseyi* infestation category colors (baseline—blue, medium—yellow, and high—red) and by number. Specific information for each cluster is provided in Table 1.

Information about the abundance of *C. rogercresseyi* comes from salmon farms located along three geographical regions: Region X [Los Lagos], Region XI [Aysén], and Region XII [Magallanes]. This area in southern Chile is a complex system of fjords and islands with a latitudinal gradient that begins in Puerto Montt (−41.4740, −72.9441) and ends around Dawson Island (−54.0370, −70.7149) (Figure 1). The salmon farms are operated by large and medium-sized companies. They are located 3 km from other farms and at 1–2 km from the coast. Salmon farms are licensed-organized within space estates called epidemiological neighborhoods, defined as space polygons that share similar production and environmental conditions for each group of farms. The salmonid production cycle begins with the transfer of broodstock in autumn for extraction, fertilization, incubation, and maintenance of eggs and fingerlings <10°C. Fish will be subjected to light and temperature regimens to induce early smoltification come spring. Smolted fish (weight > 100 g) are transferred to sea sites for a growing period in floating cages of up to 1 or 2 years for the harvest. Farms are monitored so that they enter either a 3-month rest period when harvesting or a 3-week health-check.

### Data

The study was developed using MS Excel data from the Specific Sanitary Program for the Surveillance and Control of Caligidosis in Chile SSPSC (SalmonChile INTESAL, Santiago, Chile), which carries out weekly surveys from January 2012 to December 2016 (5 years) of *C. rogercresseyi* abundance data among different life stages, divided into three categories, namely, juveniles (Chalimus I, II, III, and IV), mobile adults (males and no gravid females), and gravid female (egg sacs) (37). Sampling protocols at the farm level include the collection of 10 fish at random from four cages to estimate *C. rogercresseyi* abundance, date sample, year, month, farm ID, farm geographic coordinates (latitude and longitude), Chilean region, epidemiological neighborhood ID, and fish species (*S. salar, O. mykiss*, and *O. kisutch*). The database also presents weekly measurements of biomass (tons), number of fish (thousands), average weight (kg), average water salinity (parts per thousand), average water temperature (°C), chemical control counts, and chemotherapeutants (azamethiphos, emamectin benzoate, cypermethrin, deltamethrin, diflubenzuron, and none).

### Definition of *C. rogercresseyi* Infestation Levels and Data Management

According to sanitary regulations in Chile, all farms must apply chemical control to avoid the *C. rogercresseyi* threshold (gravid female abundance > 3). The gravid female stage is a reliable measurement due to the ease of identification with the naked eye in the field. Compared to other stages and repeated reports of this proxy, it can lead to an early harvest of farms (38). However, several *C. rogercresseyi* studies reported farms with abundances below the chemical control threshold (counts of 1 or 2), infested farms (counts around to the threshold [~3]), and severely infested farms (values above the threshold) (10, 11, 13, 39). Based on this information, we categorize the data of each into three different infestation levels: baseline (farms with abundance 0–2), medium (farms with abundance 2–4), and high (farms with abundance >4). The rationale behind this is to investigate the infested farms that never reached the parasitic threshold (baseline category), comparing them with those in the two higher levels of infestation observed (farms that reach the sanitary threshold and farms well above the threshold). Duplicate, aberrant, and writing errors were reviewed in order to clean the data. In addition, a weekly record of production and environmental factors was included by farm ID according to SSPSC data.

### Descriptive Analysis

The MS Excel data were imported into the R software (version 4.0.2) for analysis (40). The definition of the level of infestation of a farm (prevalence and growth rate) was estimated as a previous guide to the temporal window choice of space–time analysis (plotting the temporal pattern of occurrence). The dataset was arranged according to *C. rogercresseyi* threshold abundance > 3 (positive farm or case definition). Besides, it was used to describe a period prevalence, without distinction, between old and new cases by geographical region, using the “prevalence” package to estimate confidence intervals for proportions (weekly number of active farms as abundance > 3 by study period/weekly number of active farms by study period), applying a 95% confidence level through the Wald test. Also, we explore growth rate as a measure of disease spread over time (weekly number of new farms as abundance > 3 by study period/the sum of the weeks where farms were active in the study period as a time of risk), using the “incidence” package (41) to estimate the growth rate. This package uses a simple log-linear regression model where the growth rate (*r*) is the slope of the regression form log(*y*) = *r* × *t* + *b*. Due to the observation of a peak in new cases from February to August 2013, we split the data to generate the growth rate before and after the peak.

### Space–Time Cluster Analysis

A retrospective multinomial, spatial, and temporal scan test was implemented to identify farm clusters of either category of *C. rogercresseyi* infested farms: baseline (abundance 0–2), medium (abundance 2–4), and high (abundance >4). According to the baseline category and their statistical significance, clusters were prioritized for optimal *C. rogercresseyi* management. In the multinomial scan test, each observation is a case, and each case belongs to one of the three categories, evaluating whether there are any clusters where the distribution of cases is different from the rest of the study region as follows: let *Cik* be the number of observations in *C. rogercresseyi* abundance status *k* for each farm *i* (42). The probability of being in category *k* was calculated for each farm, and likely circular-shape clusters were identified using a likelihood ratio test. The likelihood function for the multinomial model is written as

$$\begin{array}{l} L\left(Z,\;p1,\;\ldots ,\;pk,q1,\;\ldots ,qk\right)\;\propto \overset{K}{\underset{k-1}{\prod }}\;\left(\underset{i\in Z}{\prod }\;pk^{Cik}\underset{i\notin Z}{\prod }\;qk^{Cik}\right), \end{array}$$

where *pk* and *qk* were the probability of being in category *k* within and outside window *Z*, respectively. To evaluate the statistical significance of the most likely cluster, Monte Carlo simulations (*n* = 999) were run. A previous analysis of sources of sea lice parasitic stages for salmon farms in Chile suggests that the exposure potential from neighboring farms is within a 30-km radius (43). Therefore, the spatial analysis window was limited to 30 km of seaway distance, whereas the temporal window was 6 months. The space–time analysis was performed using SaTScan TM free license version 9.6 (www.satscan.org). Maps were constructed using open source QGIS Geographic Information System version 2.18 (www.qgis.org).

### Multinomial Logistic Model for Associated Factors

Using a multinomial logistic regression model, production and environmental factors of the farms located within each infestation category of *C. rogercresseyi* (medium and high) were compared with those of farms located within the baseline category. This type of model is an extension of generalized linear models, allowing for an estimation of an unordered categorical response. The multinomial probability distribution is an extension of the binomial distribution. However, the multinomial logistic model tests the probability of being in a given category compared to other categories, where the relationships are thought of as relative risk ratios (RRR) (44). Production factors used as predictors were time (5 years: 2012–2016), average weight (kg), biomass (tons), number of fish (integer value), epidemiological neighborhood (administrative farms group), company (nominal name), species of fish (Atlantic salmon, rainbow trout, and coho salmon), number of chemical treatments (integer counts), and chemical treatment ingredient (azamethiphos, emamectin benzoate, cypermethrin, deltamethrin, and diflubenzuron). Environmental factors used as predictors were temperature (degrees centigrade), salinity (part per thousand), farm locality (longitude and latitude in decimal degrees), and geographical region of Chile (X, XI, and XII).

Collinearity among continuous predictors (time, average weight, biomass, number of fish, and number of chemical treatments) was assessed with pairwise scatterplot and Pearson correlation coefficients (45). We imported the MS Excel data into R statistical software v.3.6.0 (R Development Core Team) for the selection and validation of the model and then retained statistically significant factors in the final model. The multinomial model tests the probability or risk (thought of as RRR) of being in a given category or level compared to other categories as follows:

$$\begin{array}{l} \ln\frac{p\left(Y=k\right)}{p\left(Y=baseline\;category\right)}=\beta_{0}^{k}+\beta_{j}^{k}X_{j}, \end{array}$$

where the coefficients $\beta_{0}^{k}+\beta_{j}^{k}$of the *K* – 1 category were estimated based on the baseline infestation category. The model selection was carried out using the Akaike Information Criterion. The comparisons of the models were carried out by estimating the likelihood-ratio test (LRT) (*p* < 0.05). Furthermore, the explanatory power of the final model was assessed by pseudo-R2. The multinomial logistic model was performed using the “nnet” package.

## Results

### Data Exploration and Descriptive Analysis

**Figure 2:**
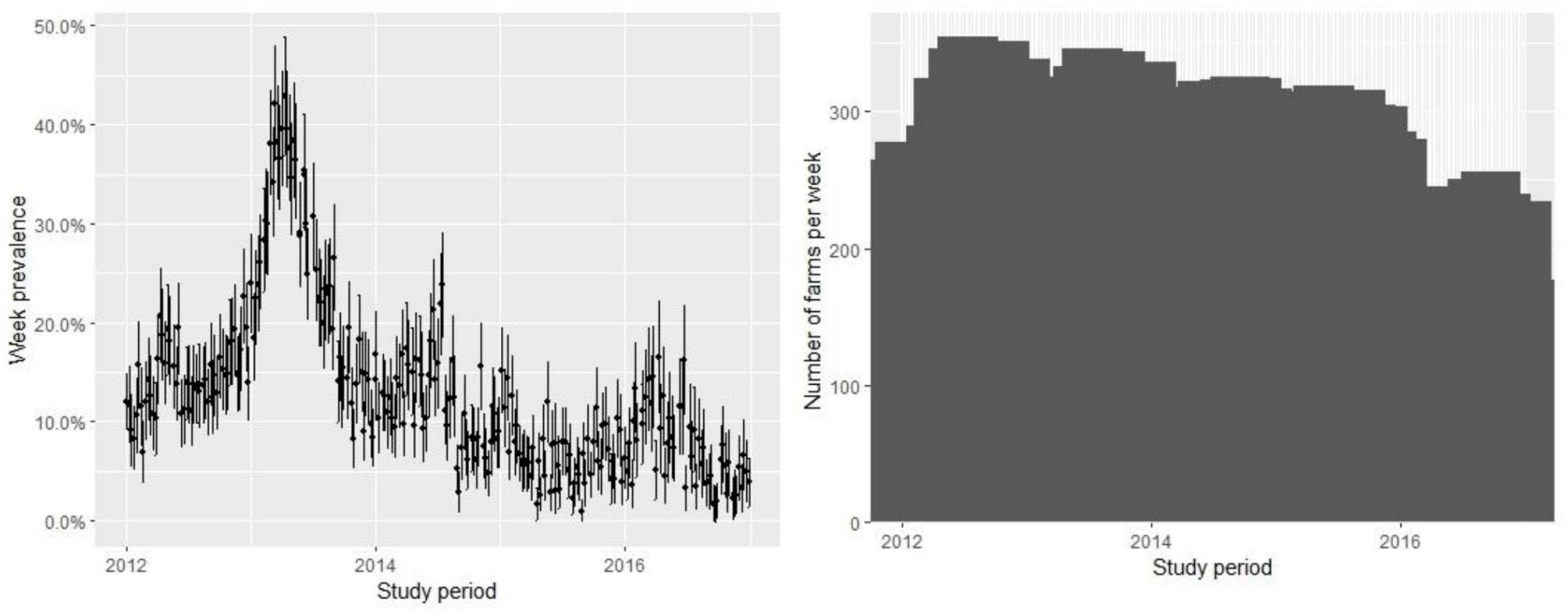
Prevalence of farms with *Caligus rogercresseyi* abundance > 3 and 95% CI (left) and measure of active farms (right) during 2012–2016 in southern Chile. The pattern suggests an epidemic curve of prevalence and the number of farms varied with relative stability during the study period.

**Figure 3:**
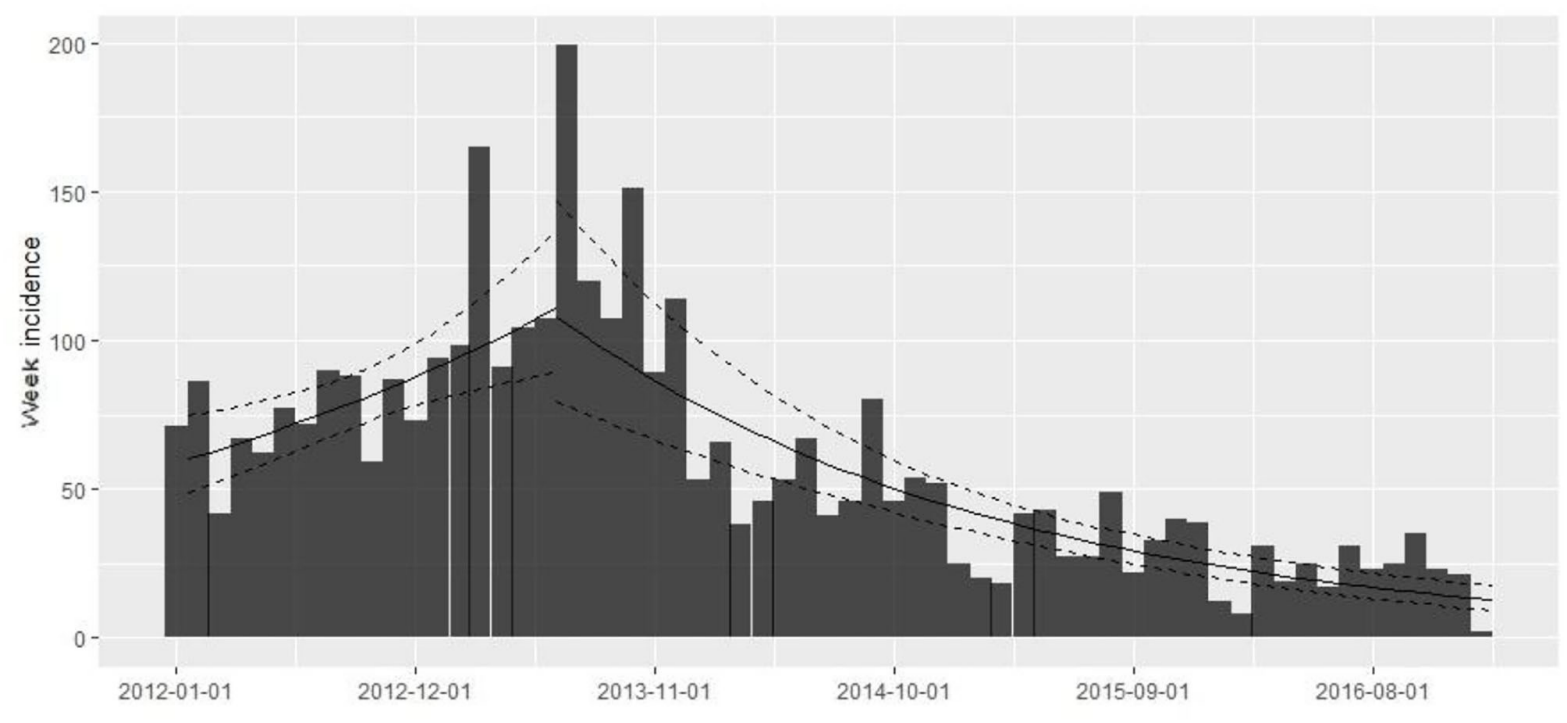
*Caligus rogercresseyi* growth rate during 2012–2016 in southern Chile (weekly number of new farms as abundance > 3 by study period/the sum of the weeks where farms were active in the study period as a time at risk) using a simple log-linear regression model where the growth rate (*r*) is the slope of the regression form log(*y*) = *r* × *t* + *b*. The rising trend line shows the growth rate before the 2013 peak (0.001 per farm-year at risk) and the descending trend line shows the growth rate after the peak (−0.004 per farm-year at risk).

The database contains 67,909 observations corresponding to weekly samples of *C. rogercresseyi* abundance from 784 salmon farms from three Chilean regions (Region X [Los Lagos], Region XI [Aysén], and Region XII [Magallanes]) for 5 years (2012–2016). Region X presents 331 farms, Region XI maintains 397 farms, and Region XII presents 58 extant farms. The weekly number of farms varied during the study period but remained relatively stable from 2012 to 2015 (mean = 277, sd = 30.67) and declined only for 2016 (mean =192, sd = 27.97) (Figure 2). The descriptive analysis and graphical tools suggest a spatial and temporal pattern in the prevalence and the growth rate (the definition of the case is at *C. rogercresseyi* abundance > 3). The prevalence of *C. rogercresseyi* increased from 11.69% (95% CI: 7.82, 15.56%) at the beginning of the epidemic in February 2013 to 30.20% (95% CI: 24.90, 35.51%), with a peak of 42.85% (95% CI: 36.91, 48.80%) in April 2013. After the peak, *C. rogercresseyi* prevalence fell to 8.33% (95% CI: 5.91, 12.04%) in December 2013, staying below 25% with two peaks of 23.79% (95% CI: 18.49, 29.08%) in May 2014 and 16.46% (95% CI: 10.78, 22.13%) in April 2016 (Figure 2). Incidence estimates considered 3,612 new cases (case per time unit [week]) during 5 years of observation, with a peak in April 2013. The growth rate before the peak is 0.001 per farm-year at risk, and that after the peak is −0.004 per farm-year at risk (Figure 3). Omitting the peak of new cases, the growth rate during these 5 years is −0.002 per farm-year at risk.

### Space–Time Cluster Analysis

The multinomial space–time scan test identified 26 significant clusters (*p* < 0.05), of which approximately half (12/26) correspond to the baseline category, whereas one is a medium infestation cluster and 13 are high infestation clusters. The number of farms per cluster varied from 4 to 39 (mean = 20, sd =10.72, Figure 1). The most likely cluster that included farms at the baseline category had a radius of 29.44 km, was located in Puerto Cisnes (−44.5725, −72.7172), and occurred from January to June 2013, during the peak of the occurrence pattern (Table 1, Figure 1). The medium infestation cluster was detected in the middle of the first year of study from July to December 2012, with a radius of 28.08 km, and it was located in the northwestern portion of National Park Laguna San Rafael (−45.7675, −74.5672) (Table 1, Figure 1). The high infestation cluster was detected during the peak of the occurrence pattern from January to May 2013, with a radius of 17.50 km, and it was located in the north area of Chiloe Island (−42.3197, −73.2691) (Table 1, Figure 1). The temporality and locality of the significant clusters are presented in Table 1. Most of the clusters coincide with the results of the descriptive analysis with an estimated peak during 2013.

**Table 1:** Clusters of *Caligus rogercresseyi* detected and their risk ratios from 2012 to 2016 in Region X [Los Lagos], Region XI [Aysén], and Region XII [Magallanes] of Chile.

			**Temporal distribution**			**Spatial distribution**				
**Category**	**Cluster**	***n***	**Start**	**End**	**No. month**	**Latitude (S)**	**Longitude (W)**	**Radius (Km)**	**Risk ratio**	***P***
Baseline (abundance 0–2)	3	41	January 1, 2012	June 30, 2012	6	41.6713	72.4222	28.75	1.99	0.001
	4	39	January 1, 2013	June 30, 2013	6	44.5725	72.7172	29.44	2.03	0.001
	7	29	March 1, 2014	August 31, 2014	6	45.4216	73.0222	28.04	2.03	0.001
	9	20	November 1, 2012	April 30, 2013	6	42.3144	72.5119	19.09	1.98	0.001
	12	15	January 1, 2014	June 30, 2014	6	46.1208	73.4502	26.50	2.02	0.001
	15	12	April 1, 2012	August 31, 2012	6	42.5200	72.7075	19.36	2.02	0.001
	20	12	October 1, 2013	March 31, 2014	6	42.9591	72.8102	29.61	2.02	0.001
	22	13	January 1, 2012	June 30, 2012	6	54.1452	71.2166	28.36	2.02	0.001
	23	3	August 1, 2012	January 31, 2013	6	43.7413	73.0097	2.34	2.01	0.001
	24	12	April 1, 2016	September 30, 2016	6	52.6286	72.3722	28.22	2.01	0.001
	25	17	July 1, 2016	December 31, 2016	6	51.9702	72.9463	24.54	2.01	0.001
	26	4	February 1, 2014	July 31, 2014	6	54.4833	71.4780	26.65	2.01	0.001
Medium (abundance 2–4)	19	20	July 1, 2012	December 31, 2012	6	45.7675	74.5672	28.08	2.15	0.001
High (abundance >4)	1	30	January 1, 2013	June 30, 2013	6	44.5980	74.0522	28.15	4.78	0.001
	2	34	March 1, 2013	August 31, 2013	6	45.2186	73.6819	26.64	3.85	0.001
	5	29	February 1, 2013	July 31, 2013	6	45.6766	73.8677	25.68	4.29	0.001
	6	24	January 1, 2013	May 31, 2013	6	42.3197	73.2691	17.50	3.97	0.001
	8	14	February 1, 2013	July 31, 2013	6	43.9661	73.1286	18.89	5.02	0.001
	10	16	2015/10/1	March 31, 2016	6	42.6355	73.5358	12.44	1.65	0.001
	11	8	February 1, 2013	June 30, 2013	6	43.9119	73.8250	8.66	5.30	0.001
	13	34	January 1, 2013	June 30, 2013	6	43.0636	73.5538	19.59	1.51	0.001
	14	26	February 1, 2013	July 31, 2013	6	41.8163	73.3341	23.64	3.06	0.001
	16	19	May 1, 2012	2012/10/31	6	44.2472	73.7372	13.41	3.39	0.001
	17	14	March 1, 2013	August 31, 2013	6	44.2113	74.2866	27.31	3.28	0.001
	18	21	January 1, 2013	June 30, 2013	6	44.5641	73.3641	29.25	2.80	0.001
	21	6	March 1, 2013	August 31, 2013	6	45.6566	73.2977	19.92	5.52	0.001

*Clusters are shown by number, and the location is depicted in Figure 1*.

### Regression Model for Associated Factors

**Figure 4:**
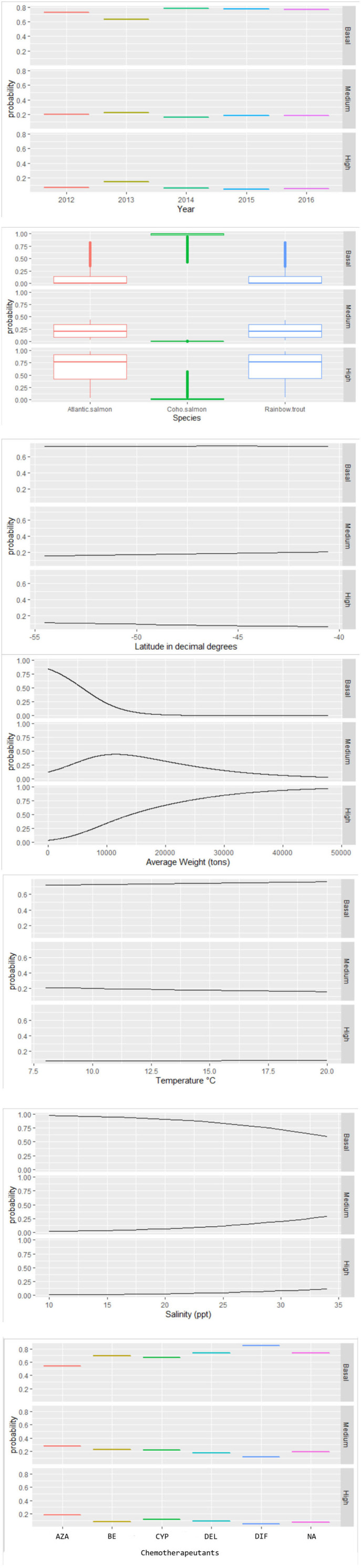
Probability values estimated by the multinomial model for the effects of associated factors to infestation category of *C. rogercresseyi* (basal, medium, and high) in southern Chile during 2012–2016. The final multinomial regression model included year category, salmon species, latitude in decimal degrees, average weight, temperature, salinity, and chemotherapeutants. *Chemotherapeutants: azamethiphos, emamectin benzoate, cypermethrin, deltamethrin, diflubenzuron, and no treatment.

There was a weak correlation between continuous predictors (the relationship by Pearson correlation coefficients was <0.7 and not significant with a *p* > 0.05), and numerical variables were log-transformed to access normality. The final multinomial regression model included year category, salmon species, latitude in decimal degrees, average weight, temperature, salinity, and chemotherapeutants. Table 2 shows the parameter estimators, relative risk ratios, standard error, and significance probability for the final multinomial model. Figure 4 shows the probability curves estimated by the model. Although the explicative power of the model was limited (pseudo-R2 = 12%), the inclusion of the variables significantly improved model fitness, comparing the LRT values of the final model (LRT = −43,566) and intercept model (LRT = −50,426). Biomass, number of fish, epidemiological neighborhood, company, the number of chemical treatments (integer counts), and geographical region of Chile lacked significance within the model.

**Table 2:** Results of the multinomial logistic model to quantify the association with production and environmental factors hypothesized to influence the *C. rogercresseyi* medium and high category in Chile during 2012–2016, compared to the baseline category as reference (year category: 2012, species: Atlantic salmon, and chemical ingredient: azamethiphos as reference values).

**Medium category Abundance 2–4**	**Coefficient**	**Std. Errors**	***z*-stat**	***P*-value**
(Intercept)	−18.100	0.590	−30.680	<0.001
2013	0.192	0.034	5.667	<0.001
2014	−0.516	0.037	−14.007	<0.001
2015	−0.395	0.037	−10.677	<0.001
2016	−0.428	0.040	−10.703	<0.001
BE	−0.400	0.103	−3.897	<0.001
CYP	−0.341	0.101	−3.367	<0.001
DEL	−0.658	0.092	−7.174	<0.001
DIL	−1.218	0.494	−2.469	0.014
NA	−0.417	0.080	−5.237	<0.001
Coho	−1.22E+01	6.10E-06	−2.01E+06	<0.001
Rainbow trout	0.263	0.027	9.663	<0.001
Temperature	−0.050	0.088	−0.571	0.568
Salinity	4.447	0.094	47.200	<0.001
Average weight	14.423	0.310	46.543	<0.001
Latitude	−0.041	0.007	−6.109	<0.001
**High category Abundance >4**	**Coefficient**	**Std. Errors**	**z stat**	***P*** **-value**
(Intercept)	−30.555	0.899	−33.997	<0.001
2013	0.861	0.048	17.760	<0.001
2014	−0.520	0.058	−9.015	<0.001
2015	−0.917	0.063	−14.465	<0.001
2016	−0.748	0.065	−11.452	<0.001
BE	−0.547	0.136	−4.013	<0.001
CYP	0.044	0.125	0.350	0.726
DEL	−0.260	0.113	−2.294	0.022
DIL	−0.743	0.745	−0.997	0.319
NA	−0.261	0.094	−2.770	<0.001
Coho	−1.07E+01	1.64E-05	−6.54E+05	<0.001
Rainbow trout	0.295	0.041	7.156	<0.001
Temperature	1.155	0.130	8.863	<0.001
Salinity	5.386	0.154	34.957	<0.001
Average weight	21.153	0.441	47.986	<0.001
Latitude	−0.149	0.009	−16.113	<0.001

*Coefficients, standard error, z-value, and significance probability are shown for each factor. Average weight, salinity, and temperature were log-transformed*.

Some different risk factors are observed in medium and high *C. rogercresseyi* abundance levels, taking the baseline category as reference (including year category: 2012, species: Atlantic salmon, and chemical ingredient: azamethiphos as reference values). Diflubenzuron and cypermethrin presented no differences in risk to higher abundance levels between diflubenzuron- and azamethiphos-treated farms (*p* > 0.05), while emamectin benzoate and deltamethrin present a statistically significant risk to farms in this category (*p* < 0.001). Besides, all chemical ingredients reduce a significant risk on the medium level of infestation (*p* < 0.001). Temperature presented a slightly non-significant risk on the medium level of infestation (*p* = 0.08), while a significant risk was observed on a high level (*p* < 0.001). In the case of the year category (increase for 2013 and decrease for subsequent years), the species of fish (rainbow trout with more risk and coho salmon with lower risk), the salinity (significant risk), the average weight (significant risk), and the latitude (lower risk), the same effect is observed in both levels of infestation.

## Discussion

Our results suggest that there is a *C. rogercresseyi* space–time pattern in southern Chile for the years 2012–2016. Furthermore, there are production and environmental characteristics that influence these patterns, depending on the infestation level (baseline, medium, and high) according to the multinomial regression analysis. This space–time pattern influenced by different factors shows a heterogeneous large-scale process of this parasite disease in an aquatic environment. The results from this study are consistent with the historical analysis suggesting that *C. rogercresseyi* infestations are clustered in particular areas, or even in particular areas at particular times (11). On the other hand, these findings suggest that management practices and sanitary regulations could vary and adapt according to the geographical location of farms across the entire area occupied by the industry. The current study also demonstrates the usefulness of space–time scan statistics (SaTScan), expressed in the identification of infestation clusters in a complex system of fjords and islands in Chile, offering a potential use as part of surveillance programs. Official notification of clusters would alert neighboring active farms to the spread of *C. rogercresseyi*, allowing them to carry out early management measures.

Space–time *C. rogercresseyi* variation in Chile has been reported before, but the cluster behavior from baseline farms concerning infestation had not been examined. Approximately 46% of clusters (12/26) present *C. rogercresseyi* abundances below the threshold of control chemical events. Some of these clusters even presented low *C. rogercresseyi* abundances during the epidemic peaks of the year 2013, as detected in the prevalence and incidence analysis (Figure 3). The estimates of the multinomial model suggest that production variable biomass, fish species, and salinity influence these baseline clusters. The density-dependent relationship between sea lice copepods and the concentration of susceptible hosts has been proposed previously (8). Our finding on the positive effect of biomass in *C. rogercresseyi* occurrence is consistent with those of different authors (1, 19, 24, 26, 43, 46–49) who suggest that it is necessary to set up a maximum level infield practice about fish density per water unit area, predisposing host susceptibility to confinement and stress increases. Bravo et al. (14) propose that a density >10 kg/m^3^ increases local *C. rogercresseyi* abundances; nevertheless, it is not clear whether this estimate can be applied to the growing trend of this economic activity. According to the probabilistic curves of our model (Figure 4), at more than 10,000 kg, farms are at risk of reaching the medium level of infestation, and at 20,000 kg of stock density, the risk of going into the high level of infestation increases (when Atlantic salmon or rainbow trout are grown). Besides, for coho salmon, the risk increases when density reaches 40,000 kg. In Chile, there are no regulations based on carrying capacity estimates to limit maximum fish biomass per area or water body (50).

According to the multinomial model, rainbow trout positively influences *C. rogercresseyi* occurrence in baseline clusters when compared to Atlantic salmon and coho salmon. Since the beginning of the industry in Chile, a greater abundance and prevalence of *C. rogercresseyi* adult stages in rainbow trout has been observed in the field, and experiment studies compared them to Atlantic salmon (11, 13, 51, 52). However, some studies have also observed similarities in *C. rogercresseyi* susceptibility by rainbow trout and Atlantic salmon, arguing differences caused by temperature > 12°C and salinity > 25 ppt (14). The susceptibility of rainbow trout to *C. rogercresseyi* could be explained by semiochemicals and mucus cell hypertrophy response (53). Besides, there is very low heritability susceptibility to *C. rogercresseyi* in rainbow trout (54). In contrast, coho salmon (as the most *C. rogercresseyi*-resistant species) has a low mucus biochemical response and a different macrophage response mechanism to *C. rogercresseyi* infestation (52).

Salinity is the environmental characteristic that reduces the occurrence of *C. rogercresseyi* in baseline clusters, giving an advantage to farms in sites with a low salinity concentration. These clusters are, spatially speaking, on the coastline, mainly in estuarine areas. This fact is consistent with the modeling of the parasitic dynamics by Mancilla-Schulz et al. (19), who compared three production sites of Region X [Los Lagos] during the years 2012–2015 and observed that the site with lower salinities presented lower *C. rogercresseyi* abundances. The tolerance of *C. rogercresseyi* at low salinities is according to that reported by Bravo et al. (12), who proves a sensitivity to salinities <20 ppt. Besides, low salinities can reduce *C. rogercresseyi* survival by up to 40%. In the Northern Hemisphere, another species of sea lice (*Lepeophtheirus salmonis*) shows adult mortality at salinities <12 ppt, egg hatching problems at salinities <15 ppt, and developmental limitations of the parasitic stages at salinities <30 ppt (55). It has been mentioned that low salinity can cause the death of the copepodid (parasitic stages) interrupting the life cycle of *C. rogercresseyi* (3), which explains the importance of this environmental characteristic to maintain baseline occurrence in clusters.

The medium infestation clusters (1/26) lack significant differences concerning associated factors with the baseline category. Despite medium infestation clusters not occupying estuarine areas, the spatial isolation from external sources could be the cause of this parasitic threshold (Figure 1). Therefore, biomass management and choice of more resistant salmon species could guarantee a better performance of these medium clusters for the occurrence of *C. rogercresseyi*. On the other hand, the measures implemented by the health authority involve other alternatives, such as chemical control rotation, coordination between neighboring farms to synchronize chemical control, and the inclusion of new chemical molecules for delousing. However, *C. rogercresseyi* populations frequently exposed to chemical molecules demonstrate changes in different molecular mechanisms associated with detoxification against pyrethroids, organophosphates, and avermectins (28). This should be taken with caution, mainly because the industry cannot dispense with chemical control, and it is rather necessary to add the intervention of fish densities within farms and the densities of neighboring farms in space–time to the available control strategies. The density-dependent dynamics of *C. rogercresseyi* represent a potent negative feedback that may limit the sanitary, economic, and environmental sustainability of salmon farming in the short term (56).

Although a number of antiparasitic treatments lack an effect, different consequences of the active ingredients in them were identified. In the baseline and medium infestation levels, all active ingredients appear to have a negative effect, especially the most recent ingredient in the study period (azamethiphos). However, at the high level of *C. rogercresseyi* infestation, cypermethrin and diflubenzuron do not affect it, suggesting a loss of antiparasitic sensibility. This is consistent with those reported by different authors about the spatially structured response and resistance of pyrethroids and the broad spectrum of azamethiphos (21, 23–26, 28, 29). This makes it necessary to include spatial structure in the application of new active ingredients and even to authorize and restrict the use of active ingredients according to sensitivity bioassays in the laboratory and field studies. In addition, the use of new ingredients could be part of an exclusive application reserved for outbreaks clusters, reducing overuse and/or incorrect use. Another central aspect is to avoid unnecessary use of chemicals and respecting the time of their use according to the label (32). Currently, all authorized drugs in Chile can be applied, with rotations, in all farms that need to control the parasite.

Temperature and latitude are the factors that influence high infestation clusters (13/26). These clusters were grouped in the north of the Chiloe Island in Region X [Los Lagos] and the central zone of the inner sea of Region XI [Aysén] during 2013, coinciding with the prevalence and incidence peaks observed in this study (Figures 1, 2). The spatial pattern observed in our study is contrary to the spatial trend reported by Hamilton-West et al. (10) about how *C. rogercresseyi* occurrence should present a decreasing pattern according to the latitudinal gradient toward Region XI [Aysén]. In our study, we observe that high infestation clusters are mostly located in Region XI [Aysén]. Otherwise, our results are consistent with the spatiotemporal patterns observed for *C. rogercresseyi* resistance to chemical control. Limited effectiveness of pyrethroids against *C. rogercresseyi* has been reported in a retrospective study in two areas during 2012–2013, one located in northern Region X [Los Lagos] and the other in the central area of Region XI [Aysén] (25). However, even though we observe a heterogeneous *C. rogercresseyi* clustered pattern, there are unmeasured factors in the understanding of those processes behind the infestation patterns.

Temperature is the main environmental characteristic influencing high infestation clusters, since the reproductive output of *C. rogercresseyi*, the time of maturation for eggs, and the development of parasitic stages can be reduced in warm water (57). Besides, the life cycle of *C. rogercresseyi* remains stable at 4–17°C and is shorter in summer (January: more than 18 days) and longer in winter (July: 45 days) (3). In a comparative study between the epidemic peaks of another sea lice species, *L. salmonis*, in Norway and Scotland, it is postulated that an increase in temperature could increase the fitness of the parasitic stages (copepodid), explaining the increase of the infestation pressure within and between neighboring clusters (58). Another mechanism not considered in the present study, but one that could explain infestation pressure, is the higher current circulation in Region XI [Aysén] channels, transporting the parasitic stages within high infestation clusters (59). The use of deeper cages is a possible measure in these clusters, avoiding surface temperature and the parasitic stage, which is generally found closer to the surface of the water. However, the influence of temperature in high infestation clusters is a cause for concern, due to the climate, changing predictions like the increase of solar radiation reaching the ocean waters, and air temperature near the ocean surface (2).

The descriptive results show that there has been a notable change in *C. rogercresseyi* prevalence and incidence over the 5 years studied. A possible explanation may be the loss of sensitivity to chemotherapy, causing an increase in the number of cases during 2013. Previously, it has been suggested that efficacy was lower for pyrethroids (deltamethrin and cypermethrin) using *C. rogercresseyi* females collected from Puerto Montt during July of 2012 (60). Besides, azamethiphos was approved as a new antiparasitic option in May 2013, explaining the decrease in cases for the years 2014–2016 (27). Furthermore, a bioassay to monitor the sensitivity of *C. rogercresseyi* males and females to deltamethrin, cypermethrin, and azamethiphos during 2013–2014 suggests a spatial variability response to deltamethrin and cypermethrin due to the continuous treatments applied on farms (29).

The findings of this retrospective study suggest the importance of addressing the density-dependent aspect as a possible IPM tool in the *C. rogercresseyi* clustering infestation process. The number of susceptible individuals per unit area is a cornerstone of the transmission in infectious disease dynamics. The open system waters in the ocean salmonid production facilities present a connection with wild fish population carrying the parasite. In Chile, *C. rogercresseyi* was transmitted to farmed fish by the native Rock cod (*Eleginops maclovinus*) (61) and Chilean silverside (*Odontesthes regia*) (62). Stocking density thresholds can decrease host stress and the impact of parasitic copepods (5). Moreover, there is a spatial density function based on the distance between active farms. Sanitary and policy management should promote attention to empirical evidence about host density threshold experiments and how this evidence is connected to endemic and epidemic *C. rogercresseyi* patterns.

## Data Availability Statement

The raw data supporting the conclusions of this article will be made available by the authors, without undue reservation.

## Author Contributions

ML-L wrote the manuscript and analyzed and interpreted data. JE-D and NZ-V collected data and wrote the manuscript. DR wrote the manuscript and prepared the tables and figures. JA wrote the manuscript and interpreted data analysis. FM wrote the manuscript, conceived and planned the study, and acquired funds. All authors contributed to the article and approved the submitted version.

## Conflict of Interest

The authors declare that the research was conducted in the absence of any commercial or financial relationships that could be construed as a potential conflict of interest.

## Publisher's Note

All claims expressed in this article are solely those of the authors and do not necessarily represent those of their affiliated organizations, or those of the publisher, the editors and the reviewers. Any product that may be evaluated in this article, or claim that may be made by its manufacturer, is not guaranteed or endorsed by the publisher.
